# Migration and Malnutrition

**DOI:** 10.4269/ajtmh.20-0962

**Published:** 2021-01

**Authors:** Jason M. Nagata

**Affiliations:** Division of Adolescent and Young Adult Medicine, Department of Pediatrics, University of California, San Francisco, San Francisco, California; Institute for Global Health Sciences, University of California, San Francisco, San Francisco, California

With wavy brown hair and fuchsia lipstick, “Maria” donned an unsettling grin. At age 15 years, she had recently emigrated from Guatemala. Her mother had immigrated to California 14 years prior, when Maria was 1 year old, fleeing an abusive husband and attempting to earn money to support Maria and her brother. Maria’s maternal grandmother subsequently raised her in Guatemala City. Maria had not seen her mother for 14 years until she moved to San Francisco. The rest of her family, including her brother and grandparents, remained in Guatemala City.

With Spanish as her only language, Maria enrolled in English as a second language class in an urban high school. Her classmates teased her heavy accent and her hefty figure; as a result, she stopped eating. She lost 41 pounds since starting school 3 months earlier. Despite the family’s financial strain and struggle to afford meals, her mother caught Maria hiding uneaten food underneath her loose lavender T-shirt, within the sleeves of her jacket, or under the dinner table until she later disposed of them in a dumpster down the street from their cramped studio apartment. Maria’s calloused knuckles revealed that she used her fingers to induce vomiting several times a day. She refused to get out of bed in the morning to avoid eating breakfast, and at night, she would emerge from bed to run in place to exercise off calories.

Maria’s mother brought her to our adolescent and young adult medicine clinic, given concern for an eating disorder. After speaking with Maria alone, she revealed that she desperately missed her family in Guatemala and wanted to return as soon as possible. She missed her grandmother’s chicken tamales and could not stand the hamburgers served at school lunch. A voice constantly taunted her for appearing fat and instructed her to hide her food and purge.

Maria attended weekly medical visits with me for over a year and also found a Latina psychologist for twice-weekly family therapy sessions. As a team, we rode through lows and highs of her eating disorder. One day, after a dinner with her mother at McDonald’s, she vomited over a dozen times in a row and had to be hospitalized since her potassium became dangerously low. She required constant supervision at school and at home; otherwise, she would not eat. At school, a teacher observed her morning snack and lunch. After school, Maria accompanied her mother who cleaned homes into the evening. With regular supervision and therapy, Maria’s body and mind stabilized.

Soon after meeting Maria, I cared for another adolescent, “Lucia,” who had stopped eating for different reasons. A month prior, she watched television news reports about Immigration and Customs Enforcement home raids and became fearful that they would come to her home and separate her from her parents. She experienced nightmares where she would return home from school and her parents would be missing. She imagined them isolated and confined in decrepit detention centers. At this same time, she experienced panic attacks and became fearful of eating. At one point, she stopped eating for several days, prompting her parents to seek medical care for her as her heart rate and blood pressure plummeted. She denied any body image concerns or weight loss attempts, but nonetheless refused to eat. After a long hospitalization and intensive therapy, she, too, began on the slow road to recovery.

From these encounters with Maria and Lucia, I learned that malnutrition can come in many forms, whether it be related to food insecurity, eating disorders, or other psychosocial stressors. Eating disorders affect diverse populations across socioeconomic spectra, including immigrants. Adolescent immigrants may experience substantial psychosocial stressors, including teasing, bullying, and fears of deportation for their families. I also learned that local health is global health. One does not necessarily have to travel abroad to practice global health or work with diverse and underserved communities. My colleagues and I refer to this as “glocal health,” signifying the symbiosis of global–local health.

After completing a year of therapy and finishing her sophomore year of high school, Maria’s vital signs stabilized, and she was medically cleared to return to Guatemala for the summer. With a wide grin, she thanked us for our support and promised to eat all of her grandmother’s home-cooked foods, including her favorite chicken tamales.

